# Hemp seeds: Nutritional value, associated bioactivities and the potential food applications in the Colombian context

**DOI:** 10.3389/fnut.2022.1039180

**Published:** 2023-01-11

**Authors:** Lidia Montero, Diego Ballesteros-Vivas, Andrés Fernando Gonzalez-Barrios, Andrea del Pilar Sánchez-Camargo

**Affiliations:** ^1^Applied Analytical Chemistry Laboratory, University of Duisburg-Essen, Duisburg, Germany; ^2^Teaching and Research Center for Separation, University of Duisburg-Essen, Duisburg, Germany; ^3^Departamento de Nutrición y Bioquímica, Facultad de Ciencias, Pontificia Universidad Javeriana, Bogotá, Colombia; ^4^Grupo de Diseño de Productos y Procesos (GDPP), Department of Chemical and Food Engineering, Faculty of Engineering, Universidad de Los Andes, Bogotá, Colombia

**Keywords:** bioactive compounds, Colombian cannabis, extraction methods, hemp seed oil, phytocannabinoids

## Abstract

For many years, Colombia was one of the countries with the largest illegal cultivation of cannabis around the world. Currently, it is going through a period of transition with a new government law that recently allows the cultivation, transformation, and commercialization of such plant species. In this sense, the identification of strategies for the valorization of products or by-products from *Cannabis sativa* represent a great opportunity to improve the value chain of this crop. One of these products is hemp seeds, which are exceptionally nutritious and rich in healthy lipids (with high content of three polyunsaturated fatty acids: linoleic acid, alpha-linolenic acid, and gamma-linolenic acid), good quality protein, and several minerals. In addition, hemp seeds contain THC (tetrahydrocannabinol) or CBD (cannabidiol) in traces, molecules that are responsible for the psychoactive and therapeutic properties of cannabis. These low terpenophenolic contents make it more attractive for food applications. This fact, together with the constant search for proteins of vegetable origin and natural food ingredients, have aroused an important interest in the study of this biomass. Some bioactivities of phytochemical compounds (polyphenols and terpenoids, mainly) present in hemp seeds have provided antioxidant, antimicrobial, and anti-inflammatory properties. This review summarizes and discusses the context of hemp use in Latin-American and the new opportunities for hemp seeds culture in Colombia considering the valuable nutritional value, main functional bioactivities, and recent advances in food market applications of hemp seeds.

## 1. Introduction

The oldest knowledge about the utilization of cannabis (*Cannabis sativa* L.) comes from Asia, where cannabis has been cultivated from 10,000 to 12,000 years (1, 2). The effects of cannabis consumption in that ancient period were related to magic, medicinal, religious, and social traditions. However, the folk knowledge about cannabis, its uses, and its effects, gave rise to the inclusion of this plant as a traditional medicine to treat common diseases in the Asian culture (3). Its use was extended to India, Egypt, the Middle East, and Europe (4). There are several hypotheses about the entrance of cannabis in Europe (2). One of these hypotheses establishes that the first date related to the emergence of cannabis in Europe is in ca. 1500 BCE (5), however, there are other indicators that date of the evidence of cannabis in the Baltic region is around 7600 BCE (6). Cannabis plants arrived in Latin America in the colonial era (16th century), when the kings of Spain promoted the development of the crop of flax and hemp in their colonies. The very resistant hemp fibers were a strategic market element, especially in the production of fabrics and ropes for navigation (7). Both King Carlos I (1545) and King Carlos IV (1779) ordered viceroys and governors of their territories to plant flax and hemp, as well as enable American ports to trade products between Spain and Portugal. Nevertheless, cannabis was also used as part of the medicines offered by pharmacies and drugstores, between the end of the 19th century and the first decades of the 20th, in countries such as Mexico, Argentina, and Brazil (7). In the second half of the 19th century, some pharmacology investigations identified its psychoactive use, and it began to be perceived as a “vice” of soldiers and prisoners and linked to criminal behavior (8). For such reasons, since the 20s, criminal legislation began to be developed in Latin America where the prison was established for those who trafficked in countries such as Argentina (1919), Colombia (1939), Mexico (1920), Costa Rica (1928) and Brazil (1930) (8).

Nowadays, cannabis is well-known for its recreational uses due to the psychoactive properties related to this plant, in particular, to one specific cannabinoid named delta-9-tetrahydrocannabinol (Δ^9^-THC). Several countries like the US, Mexico, Canada, and Paraguay have changed their legislation to allow the use of cannabis for recreational purposes. However, there are varieties of cannabis, referred as hemp, that contain traces of THC. Hemp has been cultivated and used since ancient times for different purposes. The best known of such uses has been related to its utilization as fiber crop for producing textile and paper, however, other relevant applications includes its use as an ingredient in food and medicine (1, 2). This is because hemp seeds have been reported as a source of valuable nutritional compounds such as lipids (35%, being 22% of alpha-linolenic acid), protein (25%), total dietary fiber (TDF) (28%), and minerals (5.6%) (9). In the last years, hemps seeds have found many different food and cosmetic applications that are already marketed in European countries (10). Colombia, one of those countries that, recently, has changed its legislation, established the mechanisms and procedures for the industrial use of cannabis (psychoactive) in pharmaceutical and (non-psychoactive) food, beverages, and dietary supplement applications. The most recent law in this country is the resolution 227 of 2022 (11), with which Decree 811 of 2021 is given free rein, describes the regulation of the industrial use of cannabis. Besides to discuss the history of cannabis legislation in Colombia, this review aims to make visible alternatives to valorize hemp seeds as co-product of cannabis industry in Colombia and Latin America. For this purpose, the nutritional value as well as the profile of interesting primary and secondary metabolites present in hemp seeds are described in detail. Besides, in this work, the new applications and its expansion into the Latin American industry are highlighted.

## 2. World hemp seed production

**Figure 1:**
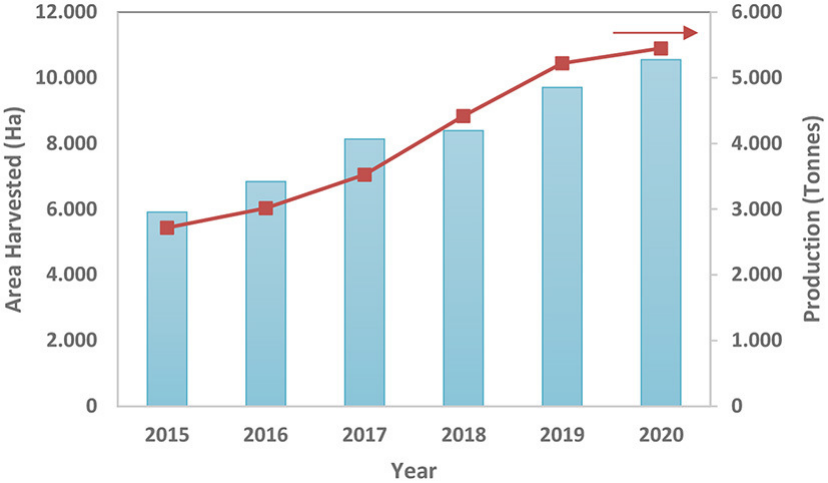
Global area harvested and production of Hemp seed (2015–2020).

Hemp seed production data are available from five countries in the FAO database for 2020 (12). Figure 1 shows the growth trends in the last years both in the cultivated area and in the production of hemp seed. Global hemp seed production values doubled from 2015 to 2020, going from 2,718 tons to 5,449 tons. This is because more and more countries have modified and focused their legislation for the cultivation of hemp, and therefore, a variety of hemp products have been developed. For 2020, Russia, Chile, and Ukraine have produced 6.866, 2.327, and 1.156 tons, respectively, being the largest producers in the world, according to the FAO database.

## 3. Hemp laws and legislation: Worldwide and Colombia

The botanical selection of cannabis contributed to increasing the yield and quality of all the cannabis by-products used for different textile, therapeutical, or food applications (13). Although the use of *Cannabis* and, in particular hemp, increased between the 13th and 18th centuries, Lavrieux et al. reported a strong decrease in pollen sediments of cannabis in the 19th century, corresponding to the global expansion of the cotton industry, which meant that cannabis lost its importance as the main European fiber crop (14).

In the last years, the legislation on industrial hemp has created a new increment in the cultivation and use of these cannabis varieties (15, 16). In Europe, between the years 1993 and 1996, its cultivation was legislated in most of the EU member states. In 2013, the European Parliament established that the cannabis varieties used for hemp production should not exceed 0.2% of THC, establishing in this way the legal use of hemp for different applications like textile, therapeutical, cosmetic, and food interests (15, 16). This novel regulation promoted, even more, the cultivation and use of hemp varieties reaching almost 35,000 ha of cultivation in 2019 (17, 18). Regarding the regulations of industrial hemp in the US, the 2014 Farm Bill established a definition for hemp and allowed its use for research and pilot programs in agriculture and university applications. In 2018, hemp was removed from the Controlled Substance Act, and it was considered as an agricultural product (19). Currently, at least 15 states and the District of Columbia allow recreational use of cannabis, and medical cannabis is legal in some ways in 30 states. As well US, Mexico, Canada, and Uruguay allow the recreational use of cannabis. Regarding its medical uses, countries such as Argentina, Australia, Barbados, Bermuda, Brazil, Chile, Ecuador, Estonia, Iceland, Jamaica, Kuwait Lebanon, Liechtenstein, Malawi, Malta, Macedonia, Norway, Panama, Paraguay, Peru, Puerto Rico, Saint Vicent and the Grenadines, Samoa, Sri Lanka, Switzerland, Thailand, The Philippines, United Kingdom, United Arab Emirates, Vanuatu, Zambia, Zimbabwe, and Colombia have recently passed laws that regulate the medicinal and therapeutic use of cannabis and its derivatives (10).

**Figure 2:**
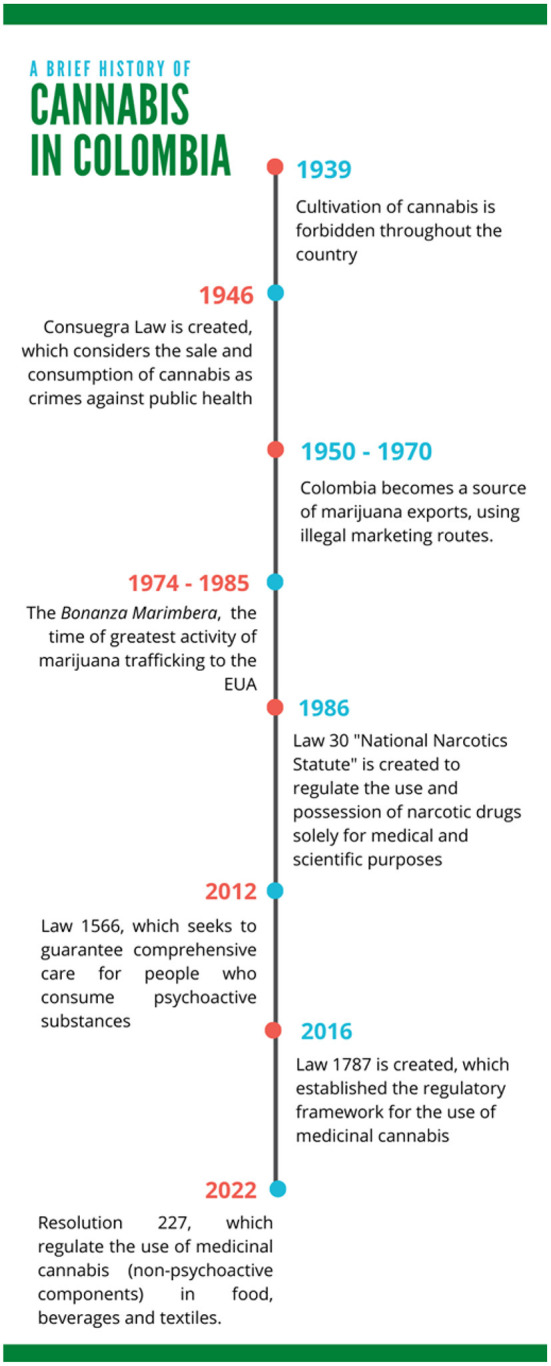
Timeline of the evolution of cultivation and consumption of cannabis in Colombia.

The history of cannabis legality in Colombia, after its pharmacology uses between the 16 to 19th centuries is described as a timeline in Figure 2. In 1939, the cultivation of cannabis was forbidden throughout the country and the destruction of existing plantations was ordered (7, 8). Subsequently, a law enacted in 1946 (called the “Consuegra Law”) toughened the penalties for the sale and use of marijuana, considering them crimes against public health (20). Despite these penal restrictions, local consumption had an important market, and between 1950 and 1970, the illegal and clandestine trade routes were strengthened in order to satisfy North American demand, becoming Colombia one of the biggest world producers, mainly from the Sierra Nevada de Santa Marta region (20, 21). In consequence, cannabis crops were spread to almost every part of Colombia, with the Caribbean coast as a principal territory of marijuana trafficking, bringing large amounts of money for this region, what was called the *Bonanza Marimbera* (20, 21). During the decades of the 70s and 80s, Colombia was greatly marked by drug trafficking (also being the main global producer of cocaine), which produced high levels of violence, promoting social and agrarian consequences that still remain. However, it was not until 1986 that the country introduced comprehensive legislation with Law 30, called the National Narcotics Statute. The purpose of this law was to regulate the production, manufacture, export, import, distribution, trade, use, and possession of narcotic drugs solely for medical and scientific purposes (22). Since the 1990s, Colombia has moved between the decriminalization of consumption and prohibition (21). In 2010, the government of Juan Manuel Santos reached a milestone in relation to drug policy, achieving an important change in the punitive approach that had been followed until then. This was motivated by the global regulation trend regarding cannabis consumption that several countries on the continent such as Uruguay, Canada, and several states in the United States had been pursuing (20, 21). In 2012, the Colombian constitutional court clarified the decriminalization of the personal dose for recreational purposes and supported consumption as an activity protected by the right to personal identity (Law 1566). Likewise, some cases of the use of cannabis extract demonstrated the therapeutic benefits of the plant to treat cases of epilepsy, chronic pain, or nausea caused by chemotherapy, which increased the support of the population to make consumption more flexible and eliminate repressive policies (23). On December 22nd, 2015, in the second term of President Santos, the decree 2,467 was signed, in order to “regulate the cultivation of cannabis plants, the authorization of the possession of seeds for cannabis planting, the control of areas cultivation, as well as production and manufacturing processes, export, import and use of these and their derivatives, intended for strictly medical and scientific purposes,” In parallel, the Colombian Senate had been working on a draft law for the same purposes. Thus, the decree 613 of 2017 regulated the Law 1787 of 2016, which allowed safe and informed access to the medical and scientific use of cannabis and its derivatives in the Colombian national territory. According to the statistics of the Ministry of Justice and Law of Colombia, from January 2017 to December 2021, 2,233 cannabis use licenses have been issued, where 262 correspond to the use of seeds for sowing, 796 for the cultivation of psychoactive cannabis plants, and 1,175 for non-psychoactive applications (24). Finally, last February, the president of Colombia, announced the approval of Resolution 227 of 2022 (11), that will regulate the use of medicinal cannabis (non-psychoactive components, such as hemps seed) in food, beverages and dietary supplements. This new law encourages the creation of new great challenges and alternatives for the recovery of cannabis products and their derivatives. Therefore, to follow, this review seeks to describe the interesting properties of hemp seed as a strategy to recognize all the opportunities that the global industry and academic community, and especially Colombian, can develop.

## 4. Nutritional value (macro and micronutrients) of hemp seed

**Figure 3:**
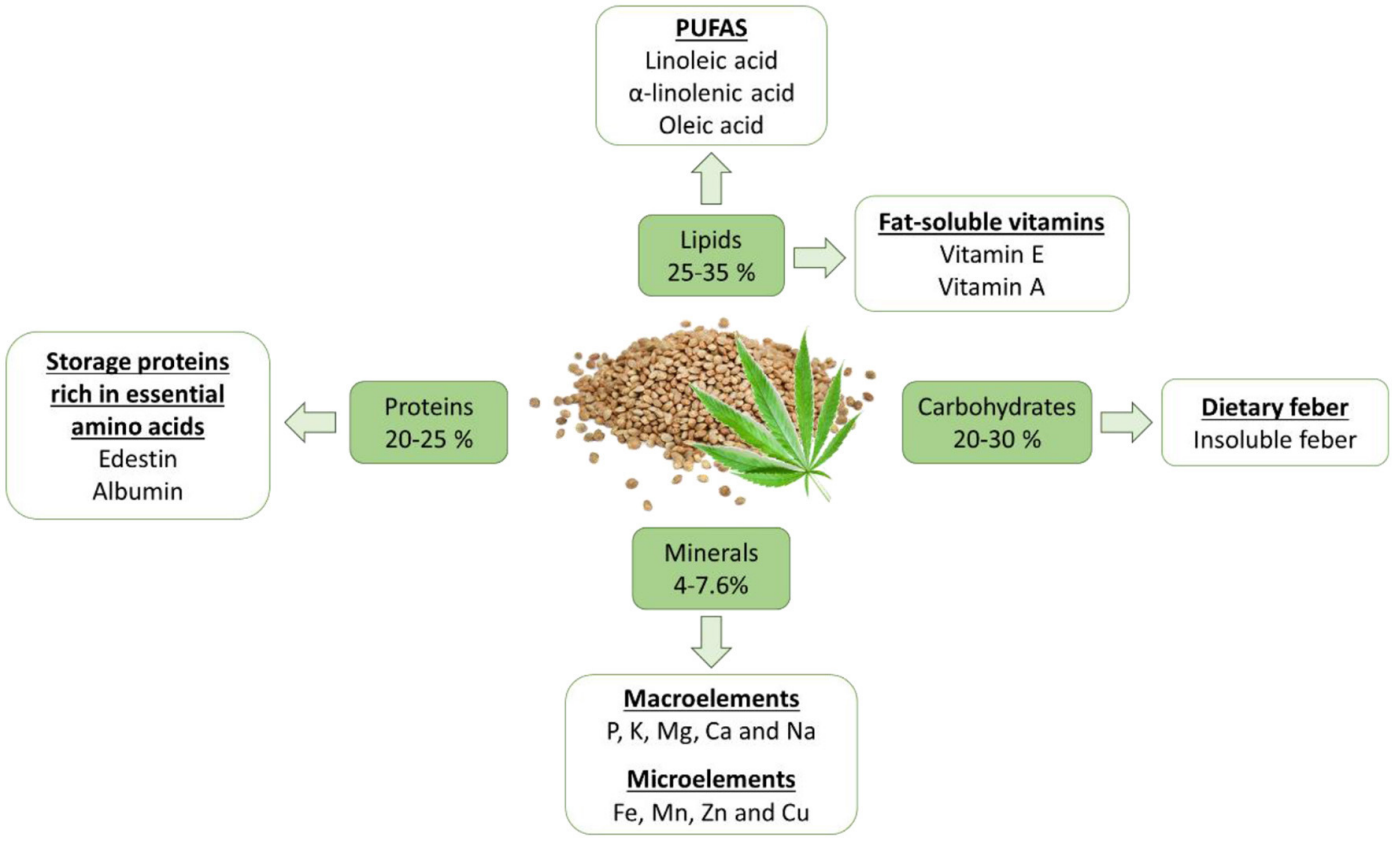
Macro and micronutrient nutritional value of hemp seeds.

The nutritional value of hemp seeds and their processed products (flour, oil, meal) has been widely studied in the last years due to the high nutritional quality of these products in terms of lipids, proteins, fiber, minerals, and vitamins (25–29). Traditionally, these products were considered for fiber production and mostly for animal feeding (25). However, nowadays hemp seeds can be considered a balanced and complete food with good nutritional characteristics that brings a positive impact on human health. The influence of hemp seeds in the food market is achieving important and growing potential. Due to the importance of their nutritional value, in this review, we have focused the attention on the macro and micronutrients present in this relevant part of the hemp plant. The main variables that affect the composition of the hemp seeds are the processing of the seeds (hulled seeds or kernel), the type of product (oil, flour, or protein powder), and the cannabis genotype and phenotype caused by environmental growing factors. Based on these features, hemp seeds present variability in their nutritional composition, and physicochemical and sensorial properties. Besides this variety, the ranges of each nutrient are well-defined. The hemp seeds content 25%−35% of lipids, 20%−25% proteins, 20%−30% of carbohydrates, and 4%−7.6% of ash (25–27) (Figure 3).

### 4.1. Lipids

One of the main reasons why hemp seeds are increasing their interest is related to their lipid content and the quality of this lipid fraction related to the fatty acid composition. More than 90% of the fatty acids present in hemp seed oil are formed by unsaturated fatty acids with an exceptional ω-6/ω-3 ratio (27, 28). Many studies have evaluated the fatty acid composition of the hemp seeds, and all of them report a high content in essential fatty acids, which are needed for the smooth functioning of the body (the human metabolism is not able to produce) and should be incorporated from the diet. Among the 90% of unsaturated fatty acids that make up the total fatty acid content of hemp seed oil, up to 70%−80% are polyunsaturated fatty acids (PUFAS). The main essential fatty acid is linoleic acid (LA; 55.1%−63.7%), followed by α-linolenic acid (ALA; 15.2%−26.2%) and by the monounsaturated fatty acid oleic acid (OA; 9%−22.5%) (30–36). These unsaturated fatty acids are related to protective effects against cardiovascular diseases, obesity, diabetes mellitus, and anti-inflammatory disorders (37). Regarding the ω-6/ω-3 ratio, EFSA establishes the optimal value between 3:1 and 5:1. The values reported in the literature for the hemp seed oil are 2.5–3.5:1, which are desirable values associated with the reduction of chronic disease risk and mortality (25, 27, 28).

The effect of the genotype and environmental factors on the composition of lipids and, in particular, fatty acids, has been widely studied (32, 34, 38–41). All the studies concluded that fatty acid composition is highly influenced by the genotype. Irakli et al. (39) reported that the most affected fatty acids by the genotype are ALA and oleic acid, while LA is less affected by the genotype variation. However, Galasso et al. (38) besides the variability of ALA, observed a genotype variability in LA as well. However, the climate and geographical area also influence the fatty acid composition. Taaifi et al. evaluated the composition of hemp seed oil of two varieties of hemp grown in four different regions from Morocco and they could determine that the observed variability in the composition was not only related to the cultivar variety but also to the growing area and the interaction of the climatic conditions (32).

Regarding the possible effect of different types of the hemp products on the fatty acid composition, Siano et al. evaluated the composition of three edible hemp sources, namely, seeds, oil, and flour for the cannabis phenotype Fedora. They did not observe significant fatty acid content differences among the three products (42). Another source of nutritional changes in the hemp seeds is the processing of the seeds. For example, roasting is a process of the hemp seeds that produces physical, chemical, structural, and organoleptic changes in the product. Babiker et al. studied the roasting time (7, 14, and 21 min) effect on the chemical composition of hemp seeds. In comparison to the effect over other nutrients, the roasting time slightly affects the composition of fatty acids of the hemp seeds. However, some changes in the fatty acid profile could be observed during the different roasting times. For instance, among the saturated fatty acids, palmitic acid was the most affected compound. On the other hand, for the unsaturated fatty acids, the roasting time of 7 min increased the concentration of OA and LA but reduced the quantity of ALA, however at 14 min the ALA was found to suffer an increase in its concentration in comparison to unroasted seeds (43).

The most popular analytical method for the analysis of fatty acids is its analysis as methyl esters (FAMES) by GC coupled to flame ionization detector (FID) using different columns and different carrier gases like helium (32, 44, 45), hydrogen (30, 34, 36, 38) and nitrogen (46, 47). Besides, another detector used for the identification of fatty acids from hemp seed oil is mass spectrometry (MS) (48, 49). Few works reported the analysis of triacylglycerols. These compounds have been analyzed in hemp seed oil using HPLC coupled to a refractive index detector (RID) using a C18 column (32). Besides, triacylglycerols from hemp seed have been also analyzed by NMR (49, 50).

### 4.2. Carbohydrates

Carbohydrates constitute between 20 and 30% of the hemp seed. Around 98% of these carbohydrates belong to dietary fiber, mainly insoluble fiber. The rest of the carbohydrates are starch; therefore, hemp seeds are considered a low-starch food matrix and a good source of fiber (25, 27).

The main fiber fraction is in the seed hull. The ratio of soluble and insoluble fiber in the hemp seed is 20:80 (31). Among the insoluble fiber, cellulose constitutes 46%, lignin 31%, and hemicellulose 22%. This insoluble fiber is an important ingredient that exerts positive functions in the human body, for example, fiber is one of the most important prebiotic compounds, and it is related to the reduction of appetite, total LDL in hypercholesterolemia and improves insulin sensitivity, therefore insoluble fiber decreases obesity and diabetes mellitus (25, 27). For these reasons, hemp seeds fiber is one of the compounds most used in food applications to fortify the fiber content of other food products, in these cases the use of the whole seed is recommended due to the fiber hull content (25). Regarding soluble fiber, a study evaluates the structure of five different hemp varieties for the use of soluble fiber for ethanol production. In this study, the concentration of glucan and xylan varied between 32.63–44.52 and 10.62–15.48%, respectively. The hydrolysis of these carbohydrates reveled a high content of glucose (63%−85%) and xylose (73%−88%) (51).

### 4.3. Proteins

Hemp seeds are a very rich source of proteins of high nutritional quality (25%−30%). However, some additional hemp seeds processes produce the concentration of the proteins like the hemp seeds defatted meal that contains around 44% of proteins, protein concentrates with 50%−75% of proteins, commonly used for food formulations, as well as hemp protein isolates (HPI) which can achieve protein concentrations higher than 85%. This last fraction has been shown to ameliorate some diseases associated with chronic pathologies like polycystic kidney or cardiovascular diseases (52). The proteins are mainly found in the internal layer of the seed, being the quantity of proteins in the hull very low (25). Besides, hemp seed protein-based products are considered less allergenic than other proteins from different edible seeds (53).

The major hemp seed proteins are storage proteins, being albumin (25%−37%) and the legumin protein called edestin (67%−75%) the main ones. These proteins are related to the regulation of organ function and human metabolism, as well as they present several bioactive peptides that have shown antioxidant and antihypertensive activities (25, 27, 52, 54). Another minor protein is the β-conglycinin (up to 5%). The hemp seed proteins content the nine essential amino acids required for the correct function of the human body. Based on the FAO/WHO, the amino acid score of the hemp seed proteins fulfills the recommendations for children between 2 and 5 years. The very high levels of arginine and glutamic acid highlight in the amino acid profile of these proteins, however, they present moderate content of sulfur-containing amino acids and a limitation in the content of lysine (55–57). The high level of arginine is especially interesting due to the important role of this amino acid in the body, for example for ammonia-detoxification, fetal growth, and reducing insulin resistance functions (27). Although albumin is considered a high-quality protein, edestin is nutritionally superior with a higher concentration of sulfur-containing amino acids (methionine and cysteine), aromatic, branched-chain, and hydrophobic amino acids (58).

Concerning the digestibility of the hemp seed proteins, no protease inhibitors have been found in the hemp seeds, which contributes to the better digestibility of the proteins (52). Mamone et al. showed a high degree of digestibility of two protein products obtained by a simple and cheap defatted process, a high purity grade hemp flour, and an HPI. Besides, they carried out a peptidomics analysis of the peptides that survive the digestion to evaluate their potential bioactivity. They detected peptides with amino acid fragments associated with bioactive sequences like bioctive peptides, which can be responsible for the reported antioxidant properties of HPI (53). Wang et al. reported a digestibility of 88%−91% of the HPI, which was higher than the soy-isolated proteins (71%) (56). The digestibility of different products derived from the hemp seeds was evaluated by House et al. They quantified the raw protein quantity and its digestibility for each of the hemp products. The results showed that the whole hemp seed presented 24% of proteins with an 84.1%−86.2% of digestibility, the dehulled hemp seed showed 35.9% of proteins and 83.5%−92.1% of digestibility, while the hemp seed meal contained 40.7 % of proteins with 90.8%−97.5% of digestibility. Therefore, the elimination of the hell fraction enhanced the digestibility of the concentrated proteins (57). In general, the protein fraction of the hemp seeds presents an excellent nutritional value, contending a good essential amino acid profile and high digestibility.

On the other hand, Sodium Dodecyl Sulfate-Polyacrylamide Gel Electrophoresis (SDS-PAGE) is very common for the analysis of hemp seed proteins (53, 55, 56, 59). A proteomic evaluation of the hemp seed was analyzed by LC-MS (MS/MS) using reversed-phase separation mode and an Orbitrap MS as an analyzer. With this method, the whole proteome of the HPI was coverage (53). Another method developed for the analysis of antioxidant and antihypertension peptides from hemp seed protein power consisted of a first purification of hemp seed protein hydrolysate by preparative LC. The active peptides were selected and analyzed by nano-LC-qTOF MS. Twenty-three short-chain peptides with bioactivity properties were identified (54).

### 4.4. Vitamins

Seeds are rich in lipids, which determine the kind of vitamins that we can find in this product. Therefore, the vitamins that are present in hemp seeds are fat-soluble vitamins, in particular tocopherols or vitamin E and vitamin A. The content of vitamins E and A in hemp seeds oil has been calculated as 562.8–929.67 and 78 mg/kg, respectively (60–62). Four different tocopherol isomers are present in the hemp seed, α-, β-, γ-, and δ-tocopherol. Among them, γ-tocopherol is the most abundant followed by α-, δ-, and β- tocopherol. γ-tocopherol is a high antioxidant compound that is responsible for the oxidative stability of hemp seed oil. On the other hand, α-tocopherol is the most vitamin active isomer. The content of α-tocopherol depends on the part of the seed, for example, the reported content in the whole seed is 0.35%−2.56%, however, if the α-tocopherol is quantified in the seeds oil, the amount can increase up to 0.7%−24.62%, depending on the genotype and environmental conditions of the plant (27).

Two analytical techniques have been reported for the analysis of tocopherols, HPLC and GC. For the HPLC analysis, different separation modes and detectors have been employed. For example, Taaifi et al. used normal phase separation (NH_2_ silica column) coupled to a diode array detector (DAD). With this method, they were able to detect and identified four different tocopherol isomers (α-, β-, γ-, and δ-tocopherol) (32). Other detectors coupled to a normal phase method reported in hemp applications are fluorescence detectors (FLD) (46) and UV-vis (33). On the other hand, reversed-phase separation modes have been used also for the analysis of tocopherols in hemp seed oils, in particular using a C18 stationary phase coupled to DAD (38), UV (34), and FLD (47), and even a C30 column coupled to FLD for the analysis of tocopherols and carotenoids (39). GC-MS analysis was applied by Aladic et al. for the characterization of tocopherols from hemp seed oil (45).

### 4.5. Minerals

Hemp seeds are also a good source of macro and microelements (4%−7.6%). The main macroelements found in the seeds are P, K, Mg, Ca, and Na, and the main trace minerals are Fe, Mn, Zn, and Cu. The quantity of these elements present in the seed also highly varies depending on different parameters such as environmental conditions, mineral soil composition, fertilizers, and plant variety. Depending on these conditions, the values reported for each element are P: 890–1,170 mg per 100 g seed, K: 250–2,821, Mg: 237–694, Ca: 90–955, Na: 6.8–27, Fe: 4–240, Mn: 4–15, Zn: 4–11, Cu: 0.5–2, and Cd: 0.0015–0.4 (25). Although other authors reported even higher values for metal elements like Fe (1,133–2,400 mg per 100 g seed), Mn (63–110 mg per 100 g seed), and Zn (42–94 mg per 100 g seed) due to the conditions of the soil of growing (40). Lan et al. reported high variability in the mineral content depending on the plant variety and crop year. They determined that the hemp flour tested was an excellent source of minerals contributing to the indicated recommended daily intake (RDI) but there were significant differences in the mineral content between the different hemp flours. Among the macro and microelements determined in the study, seven of them varied significantly in the different hemp varieties: Ca, Mg, Na, K, Fe, Mn, and Cu, while P, Mg, and Se were not affected by the variety (30).

The concentration of minerals also depends on the seed part or product. The seed flour presents a higher concentration of macroelements followed by the seeds and oil in a lower concentration. In particular, K and Ca are present in much higher concentration in the flour. In the same way, microelements are present at higher content also in the flour, except Mo which is found in major concentration in the oil. Among these trace elements, Fe is the major compound followed by Cu, Ni, and Mn (42). Another factor that affects the mineral content is the seed process like roasting. Babiker et al. determined that the roasting process decreased the content of P, Mg, and B, while the content of Ca, Fe, Cu, Mn, and Zn increased with the roasting time. They established 14 min as optimal roasting time to improve the nutritional content of the hemp seeds as well as their bioavailability was also improved due to the elimination of antinutrient compounds (43).

The composition and quantification of minerals in hemp seeds are typically carried out by inductively coupled plasma coupled to optical emission spectrometry (ICP-OES) (30, 42, 43) or by atomic absorption spectroscopy (AAS) (40).

## 5. Conventional and non-conventional extraction techniques for obtaining hemp seed oil

The most traditional processes for the extraction of hemp seed oil are cold-pressing and solvent extractions [15]. After the oil extraction, a defatted cake rich in fiber and proteins is obtained. The oil extraction yield is associated with the extraction method [16]. Cold-pressing extraction is the most simple and common process, providing extraction yields of 27%−31.5% (46). For oil extraction using organic solvents, Soxhlet using n-hexane is the most common extraction method. However, different composition of solvents has been employed in the literature (Table 1). For example, Taaifi et al. used a mixture of chloroform, methanol, and water in the proportion 2:1:1 (*v/v*) for the extraction of triacylglycerols, fatty acids, and tocopherols, identifying up to 19 fatty acids and 18 triacylglycerols (32).

**Table 1:** Extraction techniques and methods employed for the extraction of hemp seed oil.

**Hemp fraction**	**Solvent**	**Temperature (°C)**	**Time (min)**	**Extraction yield (%)**	**Ref**.
**Solvent extraction**					
Flour	Chloroform/methanol/ water (2:1:1*, v/v*)	n/r	n/r	10.4	(32)
Dehulled seed	n-Hexane/isopropanol (3:2, *v/v*)	20	60	N7	(47)
**Soxhlet**					
Seed	Petroleum benzene	n/r	300	27.8–31.9	(43)
Seed	n-hexane	70	1,440	36.30	(48)
Seed	n-hexane	n/r	480	30.6	(44)
**Hemp fraction**	**Solvent**	**Temperature (**°**C)**	**Time (min)**	**Power (W)**	**Extraction yield (%)**	**Ref**.
**Ultrasound-assisted extraction**						
Seed	n-hexane	n/r	n/r	400	35.72 (UAE + Soxhelt 37.30)	(48)
**Hemp fraction**	**Solvent**	**Temperature (**°**C)**	**Time (min)**	**Pressure (MPa)**	**Extraction yield (%)**	**Ref**.
**Pressurized liquid extraction**						
Seeds	n-hexane	100	30	6.7	24.3–28.1	(30)
**Supercritical fluid extraction**						
Seed	scCO_2_ + 10% ethanol	40	240	35	36.26	(48)
Seed	scCO_2_	40 80	n/r	30 40	22.1	(35)
Seed	scCO_2_	40	n/r	30	21.5	(44)
Dehulled seed	scCO_2_	40	240	40	40.9	(47)
Seed	scCO_2_ + 8% ethanol	76	240	30	64.45 (ω-6) 26.24 (ω-3)	(36)
**Liquefied gases**						
Dehulled seed	n-propane	60	30	10	37.81	(47)
Seed	DME	25	40	0.055	25 (unground seeds) 31 (ground seeds)	(50)

n/r, not reported.

Beside the conventional extraction methods, due to the importance of obtaining a high-quality oil, the extraction of hemp seed oil has been extracted and optimized using emergent extraction techniques like ultrasound-assisted extraction (UAE) (48, 63), microwave-assisted extraction (62), ohmic process (64), pressurized liquid extraction (PLE) (30), enzyme-assisted extraction (61) liquefied gas solvents (47, 50) and supercritical fluid extraction (SFE) (35, 36, 44, 45, 47, 48).

In a comparison between five different extraction methods, including pyrolysis, percolation, Soxhlet, UAE, and SFE using supercritical carbon dioxide (scCO_2_), the maximum extraction yield was obtained using a process consisting of a UAE extraction of the seeds using n-hexane as solvent followed by a further Soxhlet extraction with n-hexane of the UAE residue. The extraction yield of this approach was 37.3%, however, SFE should be considered as a green extraction alternative to the use of n-hexane. In that study the extraction yield achieved by SFE was 36.26%, which was similar to the maximum percentage obtained from n-hexane. Besides, the economic assessment analysis of all the tested methods revealed SFE as the most suitable extraction method (48). Ohmic extraction process has been used for the extraction of hemp seed oil using an optimal mixture of n-hexane and isopropanol in a 50:50 (*v/v*) proportion, achieving an extraction yield of 31.22% carrying out the extraction at 45°C during 60 min (64). Besides, UAE has shown to be more adequate for the extraction of hemp seed oil rich in tocopherols reducing the extraction time from 8 h to 10 min in comparison to Soxhlet extraction (63). Similar results were observed when MAE was used as extraction technique, obtaining a very rich tocopherol oil extract (929.67 mg tocopherol per kg) (62).

As mentioned above, SFE is a safe an environmentally friendly technique to avoid the use of toxic solvents such as n-hexane and tedious sample preparation steps like the removal and evaporation of solvents after the extraction. Several authors have optimized the SFE process for the recovery of high-quality oil from hemp seeds free of organic solvents. Porto et al. optimized the extraction of hemp seed oil with scCO_2_ achieving an extraction yield of 22.1% with 81% content of PUFAS (59.6% of LA, 18% of ALA) using 300 bar and 40°C as extraction conditions. In comparison to Soxhlet extraction using n-hexane the extraction yield was lower (n-hexane extraction yield = 30.6%) but the fatty acid profile was very similar (35). A combination of cold pressing extraction followed by SFE provided a high extraction yield of the hemp seed oil (33.67%) (45).

In another study, the possibility of using scCO_2_ and n-propane as liquefied gas was also evaluated. In this case, the extraction of dehulled hemp seeds was optimized using these alternative extraction processes. Comparing the extraction yield of both techniques, similar results were acquired, however, the solubility of the oil was enhanced in n-propane in comparison to scCO_2_. Besides, the extract obtained with n-propane presented a higher concentration of tocopherols and β-carotene. The extraction with n-propane allowed to perform the extraction process using lower pressure and lower quantity of solvent (47).

In general, all the SFE applications for the extraction of hemp seed oil reported similar optimal temperatures (40–70°C) and pressures (30–40 MPa). The optimization of these two parameters for the optimal extraction of oil is critical and it is important to achieve a balance since high temperatures produce and enhancement of the mass transfer ratio of analytes in the solvent, but at the same time, this increment in the temperature also reduces the density of the scCO_2_, which is associated with a decrease of the solute solubility in scCO_2_ (36, 44, 45). On the other hand, high pressures improve the extraction yield of the oil due to high pressure increases the density of the scCO_2_, and consequently, the oil solubility in the scCO_2_ is raised. Da Porto et al. described this effect for the extraction of hemp seed oil using extraction pressures up to 35 MPa, but higher pressures produced a negative impact on the extraction yield due to the highly compressed CO_2_ facilitated the solute-solvent repulsion. Besides, they described how the particle size affects the extraction yield. As expected, the smaller the particle size the better the extraction yield was observed not only due to an increment in the surface area but also because the grinding process produces rupture of the cells and helps the oil release (44). In this study, the temperature and pressure were also the most decisive parameter that affected the oil's oxidative stability. Compared to a conventional hexane extraction, the oil obtained by SFE showed better oxidative stability. This relevant aspect could be explained by the different antioxidant composition obtained with different extraction processes. Aladic et al. compared the extraction of hemp seed oil obtained by SFE, Soxhlet extraction using n-hexane, and an extraction method consisting of screw expeller pressing. The oil extracted with SFE presented the highest content of antioxidant compounds, in particular, α-tocopherol and γ- tocopherol, which could be responsible for the oxidative stability of the oil (45).

Devi et al. evaluated how the SFE parameters affected the extraction of the two main fatty acids of hemp seed oil, the ω-6 LA and ω-3 ALA. The temperature changes did not produce a big influence on the oil extraction yield, however, the pressure marked an inverse profile in the extraction of these two fatty acids with increasing temperature. They observed that the unsaturation level of the fatty acids was associated with the extraction behavior at higher temperatures. The concentration of ω-3 (more unsaturated fatty acid) was decreased while the ω-6 level (with lower unsaturations) was higher at higher temperature (36).

Dimethyl ethyl ether (DME) is another green liquefied gas that has increased its attention due to its ability to extract compounds from wet matrices with similar results to the obtained with n-hexane. DME has been used for the extraction of oil from grounded and ungrounded hulled hemp seeds. This method was simple, as well as it had low effective costs and reduced the post-extraction steps due to DME is easily separated and recovered from the extract at atmospheric pressure. In comparison to solvent extraction methods (petroleum ether and n-hexane), DME provided higher extraction yields and high oil purity. The extraction yields were 25% for the ungrounded and 31% for the grounded seeds. In this work, DME is presented as a real and good alternative for the extraction of hemp seed oil (50). Another green alternative for the extraction of bioactive compounds is the enzymatic-assisted extractions, which is a solvent-free technique. This strategy showed to be excellent for the extraction of hemp seed oil with a high concentration of tocopherols, providing a 5%−14% higher content than the control oil (61).

## 6. Bioactive compounds from hemp seeds and their potential uses in food fields

### 6.1. Phytocannabinoids

Phytocannabinoids are an interesting group of naturally occurring compounds that can produce several effects in humans by their attachment to specific human receptors (endocannabinoid system) (65). These compounds can be found in plants of the genus *Cannabis*, and these are classified into eight subgroups, including cannabigerol, cannabichromene, cannabidiol, tetrahydrocannabinol and cannabinol, cannabielsoin, isotetrahydrocannabinol, cannabicyclol, and cannabicitran (66). These phytocannabinoids are one of the most significant hemp bioactive components, because of the potential health benefits that they exert, including antineoplastic (i.e., cannabigerol, cannabidiol), antidepressant (i.e., cannabichromen), analgesic, anti-inflammatory, anxiolytic (i.e., cannabidiol), anti-epileptic (i.e., Δ9-tetrahydrocannabivarin), sleep improving and appetite-stimulating (Δ9-tetrahydrocannabinol), anti-glaucoma (Δ8-tetrahydrocannabinol), among others (67). Nevertheless, phytocannabinoids are majority biosynthesized in the glandular trichomes located all over the aerial parts of the plant in female flowers, in consequence, these compounds are not found in hemp seeds or are present in negligible amounts (68). Food products containing phytocannabinoids are mainly made by adding cannabinoid oils or extracts from hemp aerial parts (67). In any event, the phytocannabinoids content, specifically the amount of tetrahydrocannabinol, must be carefully controlled to not exceed the allowed limits as set forth by the regulations of each country. While phytocannabinoids addition could give bioactivity to food products, other important considerations are their bioavailability during/after digestion, as well as their stability during food processing (thermal, photolytic, and oxidative degradation) and storage. The recent review by Kanabus et al. (67) covered these topics in greater depth.

### 6.2. Phenolic and derivative compounds

Research on the phenolic composition of hemp seeds is not very common, and even less in hemp seed oil. However, it is well-known that the phenolic compounds present in hemp have important relevance in many of the positive effects described for this plant. Lignanamides, hydroxycinnamic acids, hydroxybenzoic acids, and flavonoids are characteristic phenolic compounds in cannabis seeds (27). Interesting reviews that describe the phenolic compound profile in hemp seed in the last years were published by Farinon et al. (25) and Leonard et al. (27).

Lignanamides are natural plant secondary metabolites derived from oxidative coupling mechanisms with hydroxycinnamic acid amides as intermediates. These compounds display powerful anti-inflammatory, antioxidant, anti-cancer, and anti-hyperlipidemic capacities *in vitro*, cell culture, and *in vivo* studies (27). Recently, Yan et al. studied the lignaamides profile from hemp seeds and isolated four new lignanamids: cannabisin M, cannabisin N, cannabisin O, and 3,3′-dimethyl-heliotropamide. They also found that some of such lignanamides compounds that have both antioxidant and acetylcholinesterase inhibitory activities are good multitarget anti-Alzheimer's disease candidates (69).

Depending on the cultivar and the part of the hemp seed, the total phenolic content (TPC) and total flavonoid content (TFC) can vary. In a general way, polyphenols are mainly located in the hull rather than in the kernel (25). According to Frassineti et al., hemp seeds cultivated in Italy presented a TPC of 2.33 ± 0.07 mg Gallic Acid Equivalent (GAE)/g dry weight (DW), and 2.93 ± 0.23 mg Quercetin Equivalent (QE)/g DW. Likewise, Vonapartis et al. (34) studied 10 industrial hemp cultivars approved for production in Canada, and reported TPC values in the range of 1.37 to 5.16 mg GAE/g. On the other hand, Smeriglio et al. (70) studied the polyphenol profile of Finola cultivar hemp seed oil, and reported a predominance of flavonones, flavonols, and flavanols, particularly naringerin (29.7 μg/100 g of FW), kaempferol-3-O-glucoside (6.5 μg/100 g of FW), and epicatechin (10.2 μg/100 g of FW), respectively. In addition, these authors found that Finola hemp seed oil had high antioxidant activity (measured by DPPH radical), inhibited β-carotene bleaching and showed high ferrous ion chelating activity. Likewise, in the Frasinetti's study on hemp seeds, an untargeted analysis of the hemp metabolites allowed the identification of caffeoyltyramine, cannabisin A, B, C as mains polyphenols (34).

### 6.3. Potential and promising applications of hemp seed and its bioactive compounds

Lately, hemp extracts and compounds have emerged as a powerful alternative in several food fields, including active packing, food preservation, functional foods, and nutraceuticals. The high development and potential of these promising applications have been driven by the wide spectrum of activities that the bioactive compounds from hemp show, such as antioxidant, anti-inflammatory, neuroprotective, prebiotic, antimicrobial, and antifungal, among others. To the best of our knowledge, most investigations in this field have been developed by European, American, and Asian researchers. The food hemp product development in Latin America is a growing field given the phytochemical profile of local hemp varieties and the legal openness about these products in the region. In this sense, Table 2 summarizes some of the most recent and representative developments on this topic as input on global trends that could be useful for the design and development of local products. As can be seen, the bioactivity of the compounds from different hemp parts has been explored. In this way, Majidiyan et al. (71) developed an active material coating using the essential oil of industrial hemp (inflorescences) reinforced in the complexation of whey protein nanofibrils and mung bean protein nanoparticles. They tested the bioactivity of this innovative coating protecting rainbow trout filets during their refrigeration storage for 14 days against lipid peroxidation (rancidity development) and psychrotrophic bacteria proliferation. Thirty volatile organic compounds composed the essential oil of the inflorescences, in particular, monoterpene and sesquiterpene hydrocarbons were the most abundant compounds. The authors suggested that the observed activity may be related to the content of α-humulene, caryophyllene oxide, and (*E*)-caryophyllene. In contrast, the sensory properties of fish filets were declining throughout the days. On the first day, the fish exhibited high sensory and quality levels, but on the 12th day, the samples did not achieve desirable scores being unacceptable for human consumption. However, the application of the hemp essential oil with carriers made of vegetable proteins can overcome the solubility limitations of bioactive volatile organic compounds from hemp in aqueous media and, in consequence, this could be used in diverse food systems. Raw and processed hemp seeds and their by-products also have been explored as sources of functional ingredients. Alonso-Esteban et al. (72) studied the chemical composition and biological activities of eight varieties (“Bialobrzeskie,” “Carmagnola,” “Fedora 17,” “Felina 32,” “KC Dora,” “Kompolti,” “Santhica 27,” and “Tiborszallasi”) of whole and dehulled hemp seeds. Samples were subjected to a maceration process using a hydromethanolic mixture as a solvent. Ferulic acid-hexoside and syringic acid were the most abundant bioactive phenolic compounds in the extracts. The extracts of whole seeds showed better antioxidant properties than those from dehulled seeds. Moreover, the dehulled seed extracts showed antibacterial activity against *Bacillus cereus, Listeria monocytogenes, and Enterococcus faecalis*. These remarkable properties could be exploited for the development of functional ingredients and food preservatives based on hemp seeds. Nevertheless, more in deep studies are needed to establish the safety of extracts and the relationship between their composition and bioactivity. On the other hand, Mikulec et al. (73) used defatted hemp flour (seeds) to substitute partially wheat flour in bread production. Hemp flour has been used to decrease gluten content in bread or to make gluten-free bakery products. In the Mukulec et al. study, the hemp flour improved the nutritional quality of bread, increasing its protein content, as well as the hemp inclusion inhibited the changes in the hardness of bread crumb and increased its browning index. Hemp flour also increased the phenolic compound's level of bread and its antioxidant activity.

**Table 2:** Bioactivities found for compounds in hemp seed.

**Hemp part**	**Activity**	**Model studies**	**Bioactive compounds**	**Extraction technique/isolation process/sample preparation**	**Potential and promising applications**	**Ref**.
Inflorescences	Antimicrobial Antioxidant	Inhibition activity against total viable and psychrotrophic bacteria, and retardation of peroxide value, thiobarbituric acid, and total volatile basic nitrogen during storage of rainbow trout filets	Volatile organic compounds [essential oil; i.e., (*E*)-caryophyllene, myrcene, α-pinene, α-humulene, terpinolene, β-pinene]	NG	Active packing Food preservative	(71)
Whole and dehulled seeds	Antioxidant	DPPH scavenging activity assay, β-carotene bleaching inhibition assay, TBARS assay	Phenolic compounds (i.e., syringic acid and ferulic acid-hexoside)	Maceration solid-liquid extraction with methanol/water 20:80 (v/v)	Functional ingredient Food preservative	(72)
	Cytotoxic	NCI-H460 cells (grown inhibition)				
	Antimicrobial	*Bacillus cereus, Listeria monocytogenes, Enterococcus faecalis* (inhibitory concentration)				
	Antifungal	*Aspergillus fumigatus, Aspergillus ochraceus, Aspergillus niger, Penicillium ochrochloron, Penicillium funiculosum, Penicillium verrucosum* var. *cyclopium* (inhibitory concentration).				
Seeds (flour)	Antioxidant	DPPH scavenging activity assay.	Phenolic compounds (i.e., ferulic acid, cinnamic acid, epicatechin, protocatechuic acid)	NG	Functional ingredient for bakery products (bread)	(73)
Seeds	Neuroprotective	Protection against hydrogen peroxide on cell damage and the expression of tyrosine hydroxylase in a cellular model (SH-SY5Y cells) of Parkinson's disease	Caffeoyltyramide	NG	Functional ingredient Nutraceutical	(74)
Seeds	Anti-neuroinflammatory activity	Inhibitory effects on TNF-α release from lipopolysaccharide-induced murine BV2 microglia cells	Lignanamide, coumaroylaminobutanol glucopyranoside, among others	Defatting with *n*-hexane, extraction using ethanol 95%, and fractionation employing different chromatographic processes	Functional ingredient Nutraceutical	(75)
Seeds	Anti-inflammatory	TNF-α and IL-6 gene expression and secretion on lipopolysaccharide-treated human primary monocytes	Phenolic acids (i.e., gallic, protocatechuic, gentisic, chlorogenic, caffeic, vanillic, *p*-coumaric, ferulic sinapic acids), flavonoids (i.e., vitexin, isovitexin, rutin, quercetin, naringenin, genistin, apigenin, diosmetin), phenolic amides (i.e., caffeoyltyramine, caffeoyloctopamine), lignamides (i.e., cannabisin A, B, and C), cannabinoids (i.e., cannabichromenic and cannabigerolic acids)	Solid-liquid extraction process from defatted (*n*-hexane) seeds followed by liquid-liquid bio-guided fractionation processes (process 1: acetone and ethanol 50%; process 2: ethanol 75%)	Functional ingredient Nutraceutical	(76)
	Antioxidant	DPPH and ABTS scavenging activity assays				
Seeds	Neuroprotective/anti-inflammatory	TNF-α, IL-1β and IL-6 gene expression and secretion on lipopolysaccharide-treated BV-2 murine cells	Protein hydrolysates	Hydrolysis by alcalase and flavourzyme	Functional ingredient Nutraceutical	(77)
	Antioxidant	DPPH scavenging activity assay and ferric reducing antioxidant power				
Seed bran (by-product)	Prebiotic	Lactic acid bacteria (LAB) fermentation (*Lactiplantibacillus plantarum* subsp. *plantarum* 98 b, *Limosilactobacillus fermentum* MR13, *Lacticaseibacillus rhamnosus* C1112) and Prebiotic activity (*Bifidobacterium bifidum* and *Escherichia coli*)	Volatile organic compounds as postbiotics produced by LAB fermentation (i.e., *p*-cymene, myrcene, eugenol)	Solid-phase microextraction	Functional ingredient	(78)
Seeds (hearts)	Antioxidant	DPPH scavenging activity assay	Lipids (i.e., linoleic acid, trilinolein, palmitic acid, oleyl oleate, γ-tocopherol, γ-sitosterol)	Ultrasound-assisted extraction using *n*-hexane, Microwave-assisted extraction using *n*-hexane, and Cold pressing	Functional ingredient Food preservative Nutraceutical	(79)
Seeds and sprouts	Antioxidant	Cellular antioxidant activity in red blood cells, oxygen radical absorbance capacity assay, and DPPH scavenging activity assay	Phenolic compounds (i.e., caffeoyltyramine, cannabisin A, B, C) and lipids (i.e., linoleic acid, linolenic acid, oleic acid)	Solid-liquid extraction by homogenization using ultraturrax and ethanol 80%	Functional ingredient Nutraceutical	(80)
	Antimutagenesis	Mutagenesis induced by hydrogen peroxide in yeast cells (*Saccharomyces cerevisiae*) D7 strain				

NG, information not given.

Anti-inflammatory and neuroprotective properties of compounds from hemp seed are one of the largest demanded bioactivities for developing functional food in preventing neurodegenerative diseases. For instance, Maiolo et al. (74) determined the ability of caffeoyltyramide, a compound isolated from a hemp seeds extract, to protect against cell damage (SH-SY5Y cells) induced by H_2_O_2_ in a Parkinson's disease model. In the same way, Zhou et al. (75) demonstrated the anti-neuroinflammatory activity on lipopolysaccharide (LPS)-induced BV2 microglia cells of a cannabisin Q and a coumaroylamino glycoside derivative isolated from hemp seeds. These compounds were able to inhibit the effects of TNF-α release from LPS-induced BV2 (1,600–6,500 pg/ml). Following a similar approach, Martinez et al. (76) identified *N*-*trans*-caffeoyltyramine as a major compound in a fraction obtained from an extract of defatted hemp seeds. This fraction reduced the inflammatory competence of LPS- treated human primary monocytes, decreasing TNF-α and IL-6 gene expression and secretion. Other bioactive compounds from hemp seeds than phenolics also exert neuroprotective and anti-inflammatory activities and could be used as functional ingredients. Rodriguez-Martin et al. (77) obtained a protein isolate from defatted hemp flour, which was hydrolyzed using alcalase serin endo-peptidase and flavourzyme leucine amino-peptidase. Protein hydrolysates (3–20 kDa) were able to down-regulate TNF-α, IL-1β, and IL-6 mRNA transcriptional levels in LPS-stimulated BV-2 microglial cells, while they also up-regulated the gene expression of anti-inflammatory cytokine IL-10. Besides the potential applications described above, an interesting proposal was developed by Nissen et al. (78), which was based on the exploitation of industrial by-products from hemp, particularly in hemp seed bran (HSB). In this study, HSB was fermented *via* lactic acid bacteria fermentation. As fermentation product, several organic volatile compounds (*p*-Cymene, Myrcene, and Eugenol) were released, which fostered the growth of probiotics (*Bifidobacterium bifidum*) while tackled enteropathogenic microorganisms (*Escherichia coli*). These results showed the high potential of HSB as functional fiber.

Due to the importance of hemp phenolic compounds in the development of new ingredients with potential health-promoting effects, the extraction of these compounds should be improved. Currently, there are two main extraction techniques to recover bioactive compounds from hemp seeds: SFE and solvent extraction (81). As already stated, SFE has mainly been employed for the obtention oils and non-polar compounds from seeds. On the other hand, the maceration process is one of the most applied strategies to extract medium-polar and polar compounds from seeds, followed by ultrasound and microwave-assisted processes (79). As can be seen in Table 2, the most employed solvents are *n*-hexane, methanol, and ethanol. These solvents have been used in single-step processes or multistep processes for the defatting and the posterior recovery of the polar fraction. Consistent with these developments, the use of GRAS solvents (i.e., water, ethanol, ethyl acetate, limonene, glycerol), design solvents (i.e., natural deep eutectic solvents), or free solvent processes on innovative and intensive extraction strategies (i.e., pressurized extraction techniques, instant controlled pressure drop, pulsed electric field, thermos-magnetic induction, flash-release) could improve the selectivity and sustainability with which bioactive compounds from hemp seeds are got, which would also improve the quality of the foods in which these compounds are used (82).

## 7. Some recent applications of hemp seeds in food products.

Uruguay was the pioneer nation to take the leap into legal cannabis production back in 2013. Likewise, other Latin American nations such as Colombia have followed the footsteps of Uruguay. Currently, 28 companies are affiliated to ASOCOLCANNA, the Colombian Association of Cannabis Industries. This association has as objective promoting, protecting, and guiding activities related to the cultivation, manufacture, and commercialization of psychoactive and non-psychoactive cannabis derivatives (83). In Table 3, these products as well as their websites for reader consulting are listed. Nevertheless, the main cannabis business models in Colombia are focused on flower production for industrial use or for exporting, the manufacture of derivatives such as extracts, distillates, and isolates from hemp for the preparation of formulations and pharmaceutical products, and the manufacture of cosmetics, dermo-cosmetics, and cosmeceutical (21).

**Table 3:** Colombian cannabis companies associated to ASOCOLCANNA (83).

**Name of cannabis company**	**Website**
Anandamida Gardens S.A.S	https://anandamidagardens.com/
Avida Global	https://www.avidaglobal.com
BG LABS S.A.S	https://bglabs.com.co/
Blueberries S.A.S.	https://www.blueberriesmed.com
Canalife	https://canalife.co
CanHuila	https://www.colgreen.com.au
Cannabis Medical Company	https://www.instagram.com/cannabismedicalco/
Colcanna S.A.S	https://www.colcanna.co/
Earth's Healing Col	https://earthshealing.org
Ecomedics S.A.S	https://cleverleaves.com/es/inicio
Econnabis S.A.S	https://www.plena-global.com
FCM Global S.A.S	https://fcm-global.com
Folium S.A.S	http://www.foliumed.com
Gaia Health S.A.S	–
Growlab S.A.S.	https://www.growlab.me
International Health Corporation	https://www.interhealthcorporation.com/
Mannta S.A.S	https://mannta.co
Medcann Colombia	https://medcann.com
Natuera	https://natuera.com
Pharmacielo Colombia	https://www.pharmacielo.com
Plantmedco	https://www.plantmedco.com
SIEMCOL	https://www.siemcol.com.co
Tarkus	https://tarkuslab.com
Varianz Bio Lab	http://www.varianz.co
Wellness Farmacéutica S.A.S	https://wellnessfarma.com

**Figure 4:**
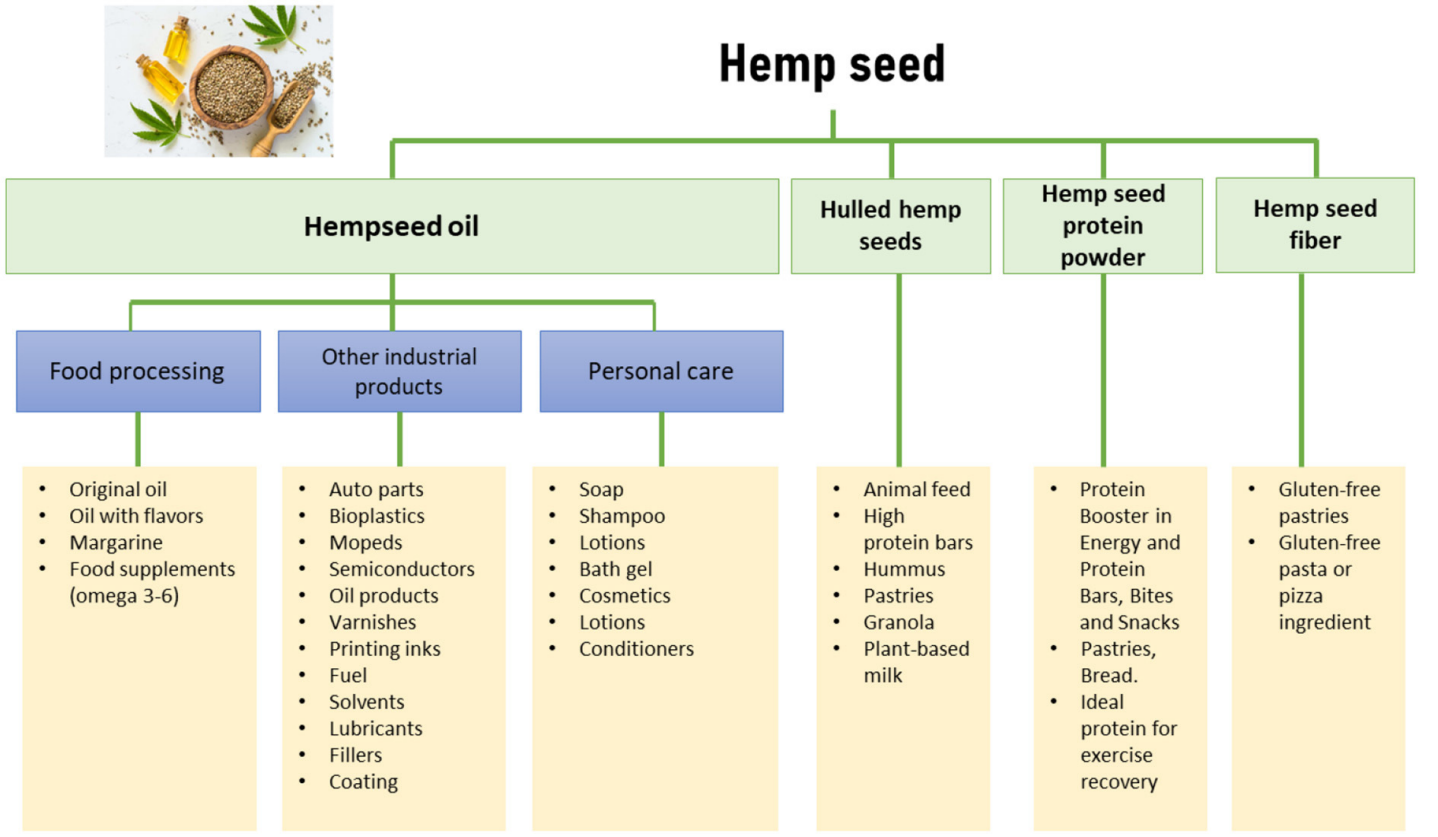
Uses and applications of hemp seed in different sectors (Modified from: New Frontier Data).

The approval of new legislation (Resolution 227 of 2022) may bring to Colombia alternatives to produce non-psychoactive (< 0.1% THC) processing products. In this sense, New Frontier Data, a data, analytics, and technology firm specialized in the global cannabis industry, has identified the uses of hemp seeds in different sectors such as mentioned in Figure 4. The hemp seed's nutritional value described above has attracted the interest of food researchers and the food industry. Different fractions such as hulled hemp seeds, hemp seed protein powder, hemp seed fiber, and hemp seed oil can be obtained from hemp seeds, and these fractions are used as new ingredients in product formulations (25). All of them can be used in the food, feed, and cosmetical field (27).

**Figure 5:**
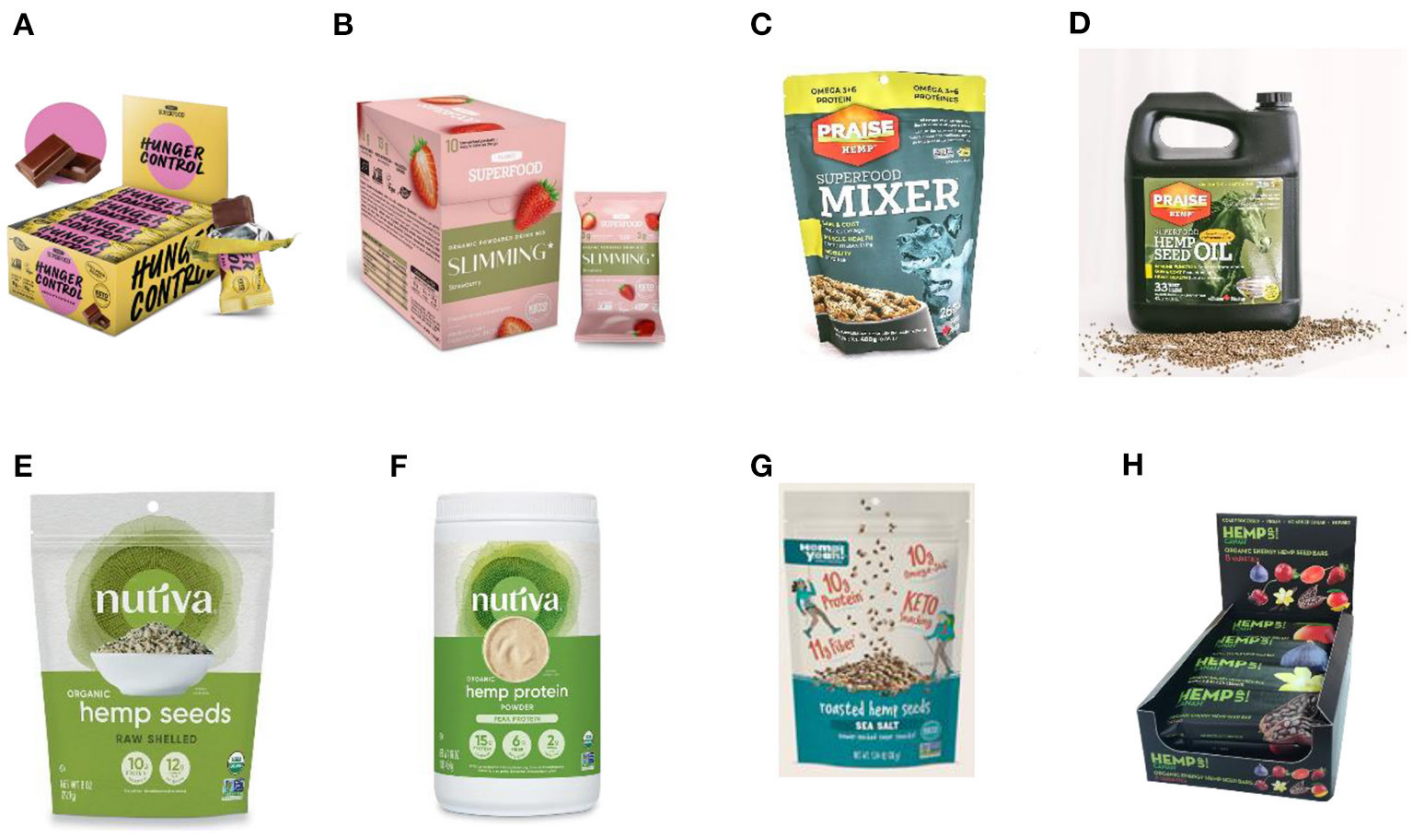
Food products derived from hemp seeds and hemp seed oil. **(A)** Hunger & craving control: vegan protein bar ^®^ (Allive^®^) **(B)**, Slimming protein smoothie: cream strawberry^®^ (Allive^®^), **(C)** superfood Mixer^®^ (Allive^®^), **(D)** Superfood Hemp seed oil 4L^®^ (Allive^®^), **(E)** Organic shelled hemp seeds ^®^ (Nutiva^®^), **(F)** Organic hemp protein powder ^®^ (Nutiva^®^), **(G)** Roasted Hemp Seeds with Sea Salt ^®^ (Manitoba Harvest ^®^), **(H)** Organic Hemp Up Energy Seed Mixed Bars 12 pieces: Sour Cherry, Mulberry, Cacao, Vanilla, Fig, Cranberry and Barberry ^®^ (Canah^®^).

There are some ventures in the world, where the focus is the use of the nutritional characteristics of hemp seeds to offer a variety of products. One of these enterprises is *Allive*^®^ (84), since, within its offer, two brands of products stand out, “planet hemp superfood” and “praise hemp”. Planet hemp superfood (85) is a recent (8 years of expertise) and innovative product line of hemp-powered superfoods. Their formulations combine plant-based protein with other nutrient-dense foods such as fruits and plants. Protein bars and smoothies are between the most sold products (Figures 5A, B). Among their products, only 100% of plant-based nutritional claims are used, and besides, they do not use genetically modified ingredients, nether gluten, or added sugar. Interestingly, these products do not contain CBD. On the other hand, “praise hemp” (86) is a line of products for horses and dogs. A blend of shelled hemp seeds and hemp seed hulls is included for formulating high protein and fiber feed (Figure 5C). Hemp oil seed is also offered as a superfood for dogs and horses with the aim to maintain a healthy amount of ω-3 and 6 fatty acids to aid in proper brain and eye development (Figure 5D). *Nutiva*^®^ (87) is another company that produces several hemp food products, among them, organic shelled hemp seed (Figure 5E), organic hemp protein powder (Figure 5F), and organic hemp seed oil can be highlighted. Shelled hemp seeds contain 33% protein by weight, along with ω-3 fatty acids and minerals. The main applications of those products are related to gastronomic uses for salads, smoothies, and sprinkled-on foods. Likewise, Nutiva's organic hemp protein powder provides high-quality plant protein with branched-chain amino acids. It is made with organic whole raw defatted hemp seeds with < 20% of oil content. The resulting seed cake is then cold milled and sifted to varying degrees to make hemp protein powder with two different levels of fiber and protein. Since 1998, *Manitoba Harvest*^®^ (88) has grown its portfolio of hemp products, having an interesting variety of hemp-based foods goods which includes shelled hemp seeds, protein powder, hemp seed oil, granola, and bars. Among their product claims, *Manitoba Harvest*^®^ stats that compared with chia and flax seeds, its product “Hemp hearts” (shelled hemp seeds) can provide two times more protein, more ω-3 and ω-6 fatty acids, 70% more iron, and half the carbohydrates per serving. Other innovative product is the “Roasted Hemp Seeds with Sea Salt” designed as snack to go for hiking, climbing, or picnic (Figure 5G). Likewise, *Canah*^®^, a German company (89), has developed also hemp feed and food products that are mixed with other foods that also are known for their high nutrition value such as blueberry, barberry, sour cherry, mulberry, cranberry, vanilla, cacao, and fig to provide enhanced bioactivity (Figure 5H).

Although that type of product is not widely used in developing countries such as Colombia, hemp seed could be a great alternative as plant-based food. As mentioned, and analyzed in this review, the complete nutritional value of hemp seed can contribute to global food necessities. According to United Nations, the growth of population will be ~10,000 million people by the year 2050 (90). Such constant growth of the population results in an increase in food production and consumption. In this sense, animal protein consumption tendencies also will increase, however, it is well-known that it causes massive deforestation, water scarcity, greenhouse gas emissions, and soil depletion that cannot promote sustainable agriculture and food production (91). So, searching for alternatives for obtaining protein from other sources is imperative. For this reason, plant-based diets have started to be practiced by many people some years ago, due to this vegetarian diet may lower blood pressure, prevent type 2 diabetes, help to lose weight, and decrease the risk of cancer, among many other benefits. In this sense, the recent interest in hemp products and the growing research work that is been made to obtain more scientific information about these interesting goods can crucially contribute to the future population problem.

## 8. Conclusions and future trends

Over the years, the use of the cannabis plant has been stigmatized in most countries of the world due to its psychoactive effects. In Colombia, the legislation for the production and consumption of this crop has undergone important changes. The history of cannabis crops has been generated from illegal traffic and social violence to the recent alternatives of medicinal products with great economic potential. This fact has aroused significant interest from the scientific and industrial community to obtain new bio-products. According to the European Industrial Hemp Association (EIHA, www.eiha.org), hemp is “the perfect crop for the circular economy of the future.” They state that hemp is a multipurpose crop with food, feed, cosmetics, construction materials, biobased plastics, textile, and energy applications. In addition, if used as an alternative to carbon-based raw materials, hemp would allow us to capture and store a substantial amount of CO_2_. One ton of harvested hemp stem represents 1.6 tons of CO_2_ absorption. On a land-use basis, using a yield average of 5.5–8 t/ha, this represents 9–13 tons of CO_2_ absorption per hectare harvested. Particularly, hemp seeds have been demonstrated to have interesting nutritional characteristics. That product is rich in high-quality proteins and has a unique essential fatty acid spectrum. With the plant-based food market growing, hemp represents the perfect source of sustainable protein to be grown locally and organically. Hemp oil and protein have been used mainly to obtain animal feed (especially dogs and horses) and food for human consumption such as shelled hemp seeds, protein powder, hemp seed oil, granola, and bars. Conventional extraction techniques such as cold pressing extraction and solvent extraction (petroleum ether and n-hexane) have been preferred for obtaining commercial hemp oil. However, other non-conventional techniques such as SFE, UAE, PLE, MAE, and liquified gases have been successful used. In addition, phytocannabinoids and phenolic compounds possess anti-inflammatory, antioxidant, and neuroprotective properties, which are very demanded bioactivities for developing functional food. Recent trends in research have been focused on the biorefinery of hemp seed for value-added products. MULTIHEMP, a project funded between 2012 and 2017 by the European Union's Seventh Framework Programme for research, technological development, and demonstration, developed an integrated hemp-based biorefinery in which improved feedstock is subject to efficient and modular processing steps to provide fiber, oil, construction materials, fine chemicals and biofuels using all components of the harvested biomass, and generating new opportunities within the developing knowledge-based bioeconomy. As a future trend, a holistic approach by the use of hemp seed could be employed as a food ingredient, in Colombia and those Latin American countries where the legislation has been relaxed. This is in the line with the increasing awareness about nutritional dietary patterns as well as the therapeutic application of plant-based food for improving the human health population, aiding to decrease nutrition-related diseases, and ensuring the physical and mental wellbeing of the population.

## Author contributions

LM: conceptualization, investigation, writing—original draft, review, and editing. DB-V: investigation and writing—original draft. AG-B: writing—review and editing and funding. AS-C: conceptualization, investigation, supervision, writing—original draft, review, and editing. All authors contributed to the article and approved the submitted version.
